# Could Physical Fitness Be Considered as a Protective Social Factor Associated with Bridging the Cognitive Gap Related to School Vulnerability in Adolescents? The Cogni-Action Project

**DOI:** 10.3390/ijerph181910073

**Published:** 2021-09-25

**Authors:** Carlos Cristi-Montero, Jessica Ibarra-Mora, Anelise Gaya, Jose Castro-Piñero, Patricio Solis-Urra, Nicolas Aguilar-Farias, Gerson Ferrari, Fernando Rodriguez-Rodriguez, Kabir P. Sadarangani

**Affiliations:** 1 IRyS Group, Physical Education School, Pontificia Universidad Católica de Valparaíso, Valparaíso 2340025, Chile; fernando.rodriguez@pucv.cl; 2 Departamento de Educación Física, Deporte y Recreación, Universidad Metropolitana de Ciencias de la Educación, Santiago 7760197, Chile; jibarramora@gmail.com; 3 School of Physical Education, Physiotherapy and Dance, Universidade Federal do Rio Grande do Sul, Porto Alegre 90040060, Brazil; anegaya@gmail.com; 4 GALENO Research Group, Department of Physical Education, Faculty of Education Sciences, University of Cádiz, 11003 Puerto Real, Spain; jose.castro@uca.es; 5 Biomedical Research and Innovation Institute of Cádiz (INiBICA) Research Unit, 11009 Cádiz, Spain; 6 PROFITH “PROmoting FITness and Health through Physical Activity” Research Group, Sport and Health University Research Institute (iMUDS), Department of Physical Education and Sports, Faculty of Sport Sciences, University of Granada, 18071 Granada, Spain; patricio.solis.u@gmail.com; 7 Faculty of Education and Social Sciences, Universidad Andres Bello, Viña del Mar 2531015, Chile; 8 Department of Physical Education, Sports and Recreation, Universidad de La Frontera, Temuco 4811230, Chile; nicolas.aguilar@ufrontera.cl; 9 Escuela de Ciencias de la Actividad Física, el Deporte y la Salud, Facultad de Ciencias Medicas, Universidad de Santiago de Chile (USACH), Santiago 9170022, Chile; gersonferrari08@yahoo.com.br; 10 Carrera de Kinesiología, Universidad Autónoma de Chile, Providencia 7500912, Chile; kabir.sadarangani@gmail.com; 11 Escuela de Kinesiología, Facultad de Salud y Odontología, Universidad Diego Portales, Santiago 8370057, Chile

**Keywords:** cognition, children, physical activity, vulnerable populations, poverty

## Abstract

The first aim was to compare differences between school vulnerability groups, fitness levels, and their combination in adolescent cognitive performance. The second aim was to determine the mediation role of fitness in the association between school vulnerability and cognitive performance. A total of 912 Chilean adolescents aged 10–14 years participated in this study. The school vulnerability index (SVI) assigned by the Chilean Government was categorized into high-, mid-, or low-SVI. Adolescents were classified as fit or unfit according to their global fitness z-score computed from their cardiorespiratory (CRF), muscular (MF), and speed/agility fitness (SAF) adjusted for age and sex. A global cognitive score was estimated through eight tasks based on a neurocognitive battery. Covariance and mediation analyses were performed, adjusted for sex, schools, body mass index, and peak high velocity. Independent analyses showed that the higher SVI, the lower the cognitive performance (F_(6,905)_ = 18.5; *p* < 0.001). Conversely, fit adolescents presented a higher cognitive performance than their unfit peers (F_(5,906)_ = 8.93; *p* < 0.001). The combined analysis found cognitive differences between fit and unfit adolescents in both the high- and mid-SVI levels (Cohen’s d = 0.32). No differences were found between fit participants belonging to higher SVI groups and unfit participants belonging to lower SVI groups. Mediation percentages of 9.0%, 5.6%, 7.1%, and 2.8% were observed for the global fitness score, CRF, MF, and SAF, respectively. The mediation effect was significant between low- with mid-high-SVI levels but not between mid- and high-SVI levels. These findings suggest that an adequate physical fitness level should be deemed a protective social factor associated with bridging the cognitive gap linked to school vulnerability in adolescents. This favourable influence seems to be most significant in adolescents belonging to a more adverse social background.

## 1. Introduction

The social environment in which children and adolescents grow up is determinant in their present and future. Poverty, low socioeconomic status (SES), social vulnerability, neighbourhood deprivation, among other adversity-related factors in childhood, are crucial predictors of mental health, income, occupational prestige, and SES later in life [1,2]. Considering the current global demographic transition where the population has increased its longevity drastically, nations have focused on reaching the full potential of children as a valuable social and economic resource [3]. Hence, cognitive functioning is a decisive and predictive success factor in life, even more than SES and intelligence quotient (IQ), being a relevant target to study in developing populations [4].

In this sense, cognitive development in childhood is a positive predictor of a diversity of socioeconomic, health, and behaviour indicators in adulthood, counteracting their social environment’s adverse impact [5]. Longitudinal studies have demonstrated that cognitive functioning at age 11 was a strong predictor of occupational prestige at age 50 [1]. Besides, children who had low inhibitory control (an executive function component) presented worse health, earned less money, were less happy, committed more crimes, and showed higher ageing signs in their bodies and brains [6,7]. It is essential to highlight that these findings were adjusted for important confounders such as IQ, personality factors, and family social background, among others. Thus, ensuring adequate cognitive development in childhood and mainly in children living in more disadvantaged social environments could be a noble strategy to reduce social disadvantage and the spread of poverty across generations [8,9].

Many strategies related to improvements in children and adolescent cognitive functioning reducing future social and health concerns [4,10,11]. Physical fitness has seemed to be a potent factor that enhances cognitive performance throughout the life cycle [12]. Increased physical fitness has been associated with better executive functions during school age (i.e., inhibitory control, working memory, etc.) [13,14,15] and predicts cognitive performance in adulthood [16]. A substantial advantage of physical fitness compared to other protective factors is that it shows mitigation of the same indicators negatively related to social disadvantages in an enjoyable, low-cost, and accessible way for all. Supporting this idea, a review addressing this field of research concluded that the most successful approach associated with cognitive performance would be one that also included emotional, social, and physical needs [4]. However, a negative secular trend in physical fitness (i.e., cardiorespiratory fitness [CRF] and muscular fitness [MF]) in children and adolescents has now been observed [17], which could exacerbate the cognitive gap caused by social inequalities and disadvantages in a sensitive age stage.

In general, there are three gaps in this field of research that are relevant to address. First, the evidence has mainly assessed how factors related to social disadvantage and physical fitness are independently associated with cognitive performance [15,18,19] or academic performance [20]. Second, most studies evaluating physical fitness include social factors (e.g., low-SES, low-income, etc.) as cofounders in their analyses [15], but the combined association between both factors (SES and fitness) has not yet been studied in depth. Finally, it is important to note that in Latin American countries, school characteristics (i.e., economic, social, and cultural status) seem to be a stronger predictor of adolescent cognitive and school performance than family SES [21].

Therefore, the primary aim of the study aim was to compare differences between school vulnerability groups, fitness levels, and their combination in adolescent cognitive performance. Whereas the secondary aim was to determine the mediation role of global fitness and its components (CRF, MF, and speed/agility fitness [SAF]) in the association between school vulnerability and adolescent cognitive performance. We hypothesise that physical fitness might show a relevant protective social role, especially within the high vulnerability group.

## 2. Methods

### 2.1. Design and Participants

This study is part of the Cogni-Action Project, which seeks to establish the associations of physical activity, sedentary behaviour, and physical fitness with brain structure and function, cognitive performance, and academic achievement in a large sample of Chilean adolescents. A detailed description of the design and methodology description was published earlier [22]. The project was approved by the Bioethics Committee of the Pontificia Universidad Católica de Valparaíso (BIOEPUCV-H103–2016) and was registered in the Research Registry (ID: researchregistry5791). This research was conducted following the Declaration of Helsinki. Written consent or assent forms were obtained before participation from school principals, parents, and adolescents.

### 2.2. Study Population

Total sample size and power calculations were based on school enrolment in grades 5 to 8 during 2016 in the Valparaiso region, reported by the Chilean Ministry of Education. A 5% maximum error, 99% confidence interval, 50% heterogeneity, and 20% drop-out rate were considered. A total of 797 participants were needed to reach representativeness. Finally, 1296 adolescents (aged 10 to 14) were involved in the Cogni-Action project due to the elevated participation and minimal exclusion criteria to maximise diversity in social, biological, and environmental influences. The present study included 912 adolescents recruited from 19 public, subsidised, and private schools after applying exclusion criteria such as being out of the stipulated age range, missing cognitive evaluation, or not having data on critical variables (i.e., fitness evaluation, covariates).

### 2.3. School Vulnerability Index

The School Vulnerability Index (SVI) was developed by the Chilean Government to measure the degree of socioeconomic vulnerability of students attending schools with partial (subsidised) or total state funding (public) [23]. The SVI considers personal and family indicators (SES, educational level of parents-guardians, students’ health status, physical and emotional well-being, and the school’s geographic location), establishing a single value for the entire establishment. SVI scores range from 0 to 100 and are updated every year. Private schools are not considered in the SVI, as they do not receive contributions from the state, so they were assigned a value of 0. Thus, the schools were classified as low (<10), middle (≥10 to <60), and high (≥60) SVI [23]).

### 2.4. Measurements 

The measurements were carried out in schools, with two visits of four hours each, separated by eight days. In the first visit, cognitive performance and anthropometry tests were taken, whereas, during the second visit, physical fitness was evaluated.

### 2.5. Global Fitness Score and Fitness Components

To compute a global fitness score, physical fitness was assessed through the ALPHA fitness test battery, which evaluates three main fitness components, CRF, MF, and SAF [24]. A z-score of each component was computed, adjusted for age and sex, and all three were added. Then, the continuous score was divided by the median, and thereby, adolescents were classified as fit or unfit according to their global fitness level. The evaluations carried out are detailed below.

### 2.6. Cardiorespiratory Fitness

CRF was evaluated with the 20-m shuttle run test and carried out at the end of the evaluation session [24]. It was performed in groups of between eight and ten participants, with a sound signal indicating the race pace, starting with 8.5 km, and increasing 0.5 km/h per minute. After covering each 20 m route, they had to wait for the next sound signal to run again. Each adolescent could withdraw when deemed necessary due to perceived fatigue or when unable to cover the distance on two occasions. Total time (in seconds) was registered, and a z-score based on sex and age was created as a normalised CRF score [25].

### 2.7. Muscular Fitness

The strength of the upper and lower limbs was evaluated as a MF indicator [24]. Upper limb strength was assessed by the maximum handgrip strength test with a dynamometer (Jamar Plus+ Digital Hand Dynamometer, Sammons Preston, Rolyan, Bolingbrook, IL, USA). The dynamometer was previously adjusted for hand size, allowing for 0 to 90 kg measures, with 0.1 kg precision. The test was performed twice (alternating both hands), standing with an extended elbow, and the best result between measurements was recorded. Then, in order to create a relative gauge of upper limb strength, the score was divided by body weight (handgrip-strength/weight).

Furthermore, lower extremity strength was evaluated by the standing long jump (SLJ) test. Adolescents stood behind the line and, at the verbal signal, jumped as far as possible, starting and landing with both feet simultaneously. This test was performed twice (with at least 1-min rest between each try), and the longest jump was recorded in centimetres (cm). Finally, the MF z-score was obtained based on the sum of the standardised values by sex and age of handgrip-strength/weight and SLJ test.

### 2.8. Speed-Agility Fitness

The SAF was evaluated by the 4 × 10 m shuttle run test [24]. This test considers movement speed, agility, and coordination. Two parallel lines were marked on the floor (5 m long) and separated by 10 m (delimited with cones). Adolescents had to run as quickly as possible, carrying a piece of cloth and dropping it on the next line (approx. 50 cm from the line). They would then pick up another piece of cloth and would repeat this sequence three times more. This test was performed twice, and the lowest time was recorded. Time was multiplied by −1, so a higher score indicated better performance. Finally, a z-score base on sex and age was created as a normalised SAF score.

### 2.9. Global Cognitive Performance

The NeuroCognitive performance test (NCPT) (Lumos Labs, Inc., San Francisco, CA, USA) was used to assess diverse tasks in order to establish a global cognitive performance score [26]. The NCPT is a brief, repeatable, web-based platform of cognitive tasks intended to measure functioning across several cognitive domains. The NCPT was applied in schoolrooms, in groups of 25 participants, each one with a laptop. The entire session lasted around one hour, which consisted of a brief explanation of the session’s objective, a demonstration and practice before each test, and finally, the execution. This study evaluated eight cognitive tasks involving the following cognitive functions: Trail Making A and B assess attention, cognitive flexibility, and processing speed; the “Forward Memory Span” and the “Reverse Memory Span” evaluate short-term visual memory and working memory; the “Go/No-Go” test, assessing inhibitory control and processing speed; the “Balance Scale,” indicating quantitative and analogical reasoning; the “Digit Symbol Coding,” valuing processing speed; and finally, the “Progressive Matrices,” assessing problem-solving and reasoning/intelligence. 

Each test was scaled following a normal inverse transformation of the percentile rank [26]. These procedures benefit from having scaled scores derived of the same normal distribution with a mean of 100 and a standard deviation of 15. Scores from the “Go/No-Go” were multiplied by −1, so a higher score indicated better performance. Finally, all eight task scores were added to compute the global cognitive score.

### 2.10. Covariates 

Four covariates were used in all analyses, such as sex, school, body mass index z-score (BMIz), and peak high velocity (PHV). Sex (1 = boys; 2 = girls) was included due to differences in maturity stage at the same chronological age between boys and girls [27], while schools are strongly associated with the SES [21]. BMIz: weight was measured with a digital scale (OMROM, HN-289-LA, Muko, Kyoto, Japan) and height with a portable stadiometer (SECA, model 213, GmbH, Hamburg, Germany). Then, the BMIz was calculated using the World Health Organization 2007 growth benchmark for school-age children [28]. PHV is an indicator of the children/adolescents biological maturity and was calculated using the equation by Moore and colleagues [27], subtracting the PHV age from the chronological age. Thus, the difference in years was defined as a value of maturity offset.

### 2.11. Statistical Analyses

Descriptive data were presented as means, standard deviation (SD), frequency, and percentage. Differences between low-, middle- and high-SVI levels in continuous and factor variables were tested using the Student’s t-test or the Chi-square test. Parametric analyses were carried out according to the central limit theorem, which indicated that this kind of test is safe with skewed data when the sample size is over 500 [29]. At the same time, Q-Q visual residual inspection was performed. For the study’s primary aim (primary outcome), three one-way analyses of covariance (ANCOVA) were performed (adjusted for sex, PHV, BMIz, and schools) to assess mean differences in children’s cognitive performance according to SVI levels, global fitness levels, and their combination. Tukey *post-hoc* pairwise comparison was conducted to establish differences in the marginal estimated means in each pair of levels (estimated mean marginal and standardised error [SE] are presented in results and 95% confidence interval [CI] in plots). Additionally, two effect size estimations were used, the first (n^2^p) for the global analysis and the second (Cohen’s d) for comparisons between levels. The n^2^p was interpreted as small at 0.01, medium at 0.06, and large at 0.14; while the Cohen’s d was interpreted as no effect (<0.2), small (0.2 < 0.5), medium (0.5 < 0.8), and large (≥0.8) [30]. Statistical analyses were performed with the free and open statistical software JAMOVI (version 1.6.7.0), and statistical significance was set at *p* < 0.05. A statistical trend was declared when one indicator was significant and the other one was not (e.g., *p* > 0.05 and Cohen’s d ≥ 0.2).

For the study’s secondary aim (secondary outcome), first, zero-order correlations among principal study variables were performed to establish their associations (Table 2). Second, multicollinearity was checked before analyses (Variance Inflation Factor [VIF]). Third, a total of 28 mediations were performed considering SVI (categorical and independent variable) as a predictor of cognitive performance (outcome, dependent variable) and adolescents’ global fitness score and its components (CRF, MF, and SAF) as mediators between both predictor and outcome. Two covariate models were implemented, “Model 1” (sex, school, BMIz, and PHV) was included when the global fitness score mediation was analysed, while “Model 2” (Model 1 plus the other two fitness components) was included when a particular fitness component was analysed (e.g., CRF as mediator adjusted for MF and SAF). Sequential multicategory analysis was used to establish mediations between SVI levels.

For each mediation, four regressions were performed, equation (a) predictor to the mediator; (b) mediator to the outcome, (c) predictor to the outcome (total effect), and (c’) predictor to the outcome, including the mediator (direct effect). A bootstrapped (5000 samples) linear regression analysis through PROCESS SPSS script [31] was performed in all regressions. The indirect effect was computed (Equation a*b), and it was considered significant when zero was outside of the 95% CI. Besides, the percentage of mediation was estimated 1-(equation c’/equation c). Mediations were categorised as (a) “Full mediation:” the indirect effect only exists through the mediator, which means the indirect effect exists, but has no direct effect, (b) “Partial mediation:” both indirect and direct effects are significant, and (c) “No mediation:” no significant indirect effect is observed.

## 3. Results

A total of 912 Chilean adolescents aged 10–14 years participated in this study, 460 boys (50.4%) and 452 girls (49.6%). Table 1 shows the participants’ characteristics. Significant differences among SVI levels were found in almost all variables except in sex. Simultaneously, we observe differences by sex in all variables except in the global cognitive performance (*p* = 0.462) and the global fitness score (*p* = 0.056).

### 3.1. Primary Outcome

**Figure 1:**
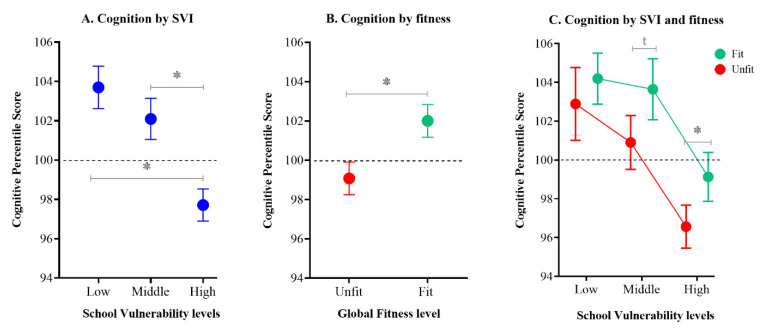
Cognitive performance according to school vulnerability and global fitness levels. (**A**) Cognitive performance according to school vulnerability index levels; (**B**) Cognitive performance according to level of global fitness; (**C**) Cognitive performance according to level of global fitness and school vulnerability index levels. Estimated mean marginal and 95% CI. Normal distribution of the cognitive percentile score with a mean of 100. * indicate *p* < 0.05; t indicates a trend (Cohen’s d > 0.2 and *p* > 0.05).

Figure 1A shows independent differences (mean ± SE) between the adolescents’ cognitive performance according to SVI levels (low-SVI: 103.7 ± 0.6, mid-SVI: 102.1 ± 0.5, and high-SVI: 97.7 ± 0.4; F_(6,905)_ = 18.5; *p* < 0.001; n^2^p = 0.087). No difference was observed between low- and mid-SVI levels (*p* = 0.098; Cohen’s d = 0.19), while significant differences were found between mid- and high-SVI levels (*p* < 0.001; Cohen’s d = 0.52) and low- and high-SVI levels (*p* < 0.001; Cohen’s d = 0.72). Figure 1B shows that fit adolescents had higher cognitive performance than their unfit peers (unfit: 99.1 ± 0.4, fit: 102.0 ± 0.4; F_(5,906)_ = 8.93; *p* < 0.001; n^2^p = 0.024; Cohen’s d = 0.34).

Figure 1C shows combined differences between cognitive performance according to SVI and fitness levels (F_(11,900)_ = 12.0; *p* < 0.001). Intra-group analyses found a statistical difference in the high-SVI group between fit and unfit adolescents (high-SVI/fit: 99.3 ± 0.7 vs. high-SVI/unfit: 96.6 ± 0.6; *p* = 0.034; Cohen’s d = 0.32) and a statistical trend between fit and unfit adolescents in the mid-SVI group (mid-SVI/fit: 103.7 ± 0.8 vs. mid-SVI/unfit: 101.0 ± 0.7; *p* = 0.125; Cohen’s d = 0.32). No difference was observed in the low-SVI group (low-SVI/fit: 104.5 ± 0.7 vs. low-SVI/unfit: 103.2 ± 1.0; *p* = 0.859; Cohen’s d = 0.16).

Inter-group comparisons found a trend between high-SVI/fit and mid-SVI/unfit levels (*p* = 0.465; Cohen’s d = 0.21). No difference was observed between adolescents from the mid-SVI/fit group and the low-SVI/unfit group (*p* = 0.999; Cohen’s d = 0.06) (Figure 1C).

### 3.2. Secondary Outcome

Table 2 shows zero-order correlation coefficients among the study’s main variables included in the mediation analysis. All variables were associated significantly; nonetheless, the SVI was negatively related to fitness scores and cognitive performance. A positive association was observed between fitness scores and children’s cognitive performance. No multicollinearity was observed (VIF range between 1.021 to 1.473).

**Figure 2:**
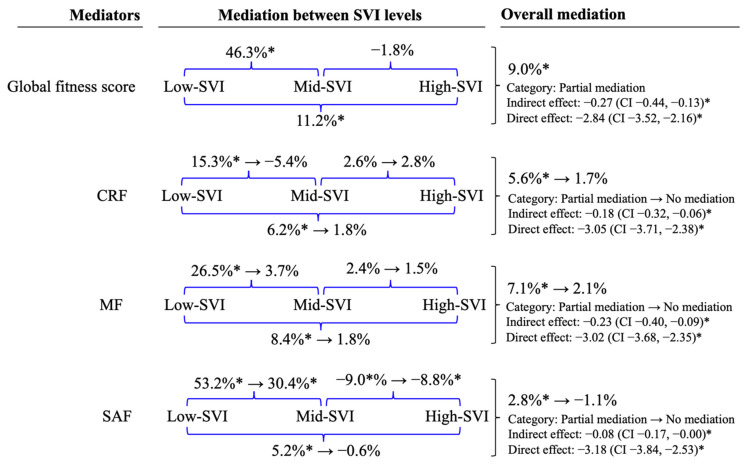
Total and specific mediation percentage and indirect effect significance between SVI levels by fitness components on adolescents’ cognitive performance. General scheme: Model 1 → Model 2; Model 1: Adjusted for sex, school, BMIz, and PHV; Model 2: Adjusted model 1 plus other two fitness components. * indicates the indirect effect is significant statistically (mediation). CRF: Cardiorespiratory Fitness; MF: Muscular Fitness; SAF: Speed-Agility Fitness.

Figure 2 shows the total and specific mediation percentage and indirect effect significance among SVI levels by fitness components on adolescents’ cognitive performance. The four fitness variables mediated the association between SVI and cognitive performance significantly (“overall mediation”), global fitness score by 9.0%, CRF by 5.6%, MF by 7.1%, and SAF by 2.8%. However, each fitness component (as mediator) lost significative mediation effect when the other two fitness components were included in model 2.

Regarding the fitness mediation effect through a sequential multicategory analysis by SVI level (“mediation between SVI levels”), the main statistical differences were found between the low- and mid-SVI and low- and high-SVI. CRF and MF lost the significant mediation effect after controlling for model 2. SAF was the only one that remained significant after adjusting to the other two fitness components (between low- and mid-SVI, and mid- and high-SVI). A detailed mediation summary as supplementary material (Table S1).

## 4. Discussion

The primary aim of this study was to compare differences between school vulnerability groups, fitness levels, and their combination in adolescent cognitive performance. Overall, independent differences showed that higher SVI was attributed to lower cognitive performance, whereas adolescents who were fit had a higher cognitive performance. The combined difference (SVI and fitness levels) allowed us to determine two main findings. First, fit adolescents belonging to the high- and mid-SVI levels had higher cognitive performance than their unfit peers. Second, fit adolescents from higher-SVI levels showed no cognitive differences with their unfit peers from lower SVI groups.

The secondary aim was to determine the role of global fitness mediation and its components (CRF, MF, and SAF) in the association between school vulnerability levels and adolescent cognitive performance. In general, mediation percentages of 9.0%, 5.6%, 7.1%, and 2.8% were observed for the global fitness score, CRF, MF, and SAF, respectively. Besides, the mediation effect was statistically significant between the low-SVI group and the other two levels (mid- and high-SVI) but not between mid- and high-SVI levels. Finally, when a fitness component was adjusted for the other two components, we did not observe any mediation, demonstrating the high interdependence among the three fitness components.

### 4.1. Independent and Combined Differences between Fitness, SVI, and Cognitive Performance

Our findings from the independent differences are in line and supported by various studies in this research field [15,32,33,34,35]. On the one hand, increased physical fitness has shown a positive influence over several cognitive skills which have been evaluated in the present study and conformed the global cognitive performance indicator, such as short-term visual memory and working memory [32], reasoning [33], processing speed [34], intelligence [35], and global cognitive performance [15]. On the other hand, children and adolescents immersed in social contexts with low household income [1,36], low parental education [36], and poverty [37] evidence lower cognitive performance. Both physical fitness and social vulnerability seem to influence cognitive skills in children and adolescents through a broad diversity of brain health markers such as depressive symptoms, cortisol levels, neuroelectric activity, brain function, and brain macro- and microstructure [12,38,39,40]. Thereby, it is plausible to speculate based on the literature and our findings concerning a possible competitive process between these two environmental factors that would be more accentuated during sensitive development stages, like adolescence [12,39]. 

To our knowledge, to date, there is no study exploring the combination of socioeconomic factors, physical fitness, and cognitive functioning in adolescents, focusing on the protective social role of physical fitness. Important to note is that the limited evidence on this area has been performed mainly in developed countries [41,42], using socioeconomic variables as covariates [43,44] and focusing on academic achievements as the primary outcome [41,42]. Only one study involving the present adolescent population showed a positive association between SVI and physical activity, and between physical activity and physical fitness (CRF, MF, and SAF) [45]. These findings point out that adolescents from schools with high vulnerability could accumulate more physical activity than their peers from a better social background; nonetheless, this favourable link could be lessened due to the inverse relationship between SVI and physical fitness [45].

Our results indirectly showed that when a fitness cut-point is established (in our case, the median on the global fitness score), it was possible to detect cognitive differences between fit and unfit adolescents belonging to the two highest SVI levels (high- and mid-SVI). Thus, a minimum level of fitness seems to be necessary to observe benefits at the cognitive level. Hence, although increasing physical activity level is encouraged in childhood to counteract the high rates of physical inactivity and sedentary behaviour [17] and, in turn, improve their cognitive profile [12], the primary outcome and strategy must be focused on increasing adolescent physical fitness [45]. Supporting this statement, many studies have shown that physical fitness is strongly associated with cognitive and academic achievements rather than physical activity alone [46,47].

Considering the time adolescents spend in schools, the low student performance on standardised tests [48,49], and the negative global trend on physical fitness [17], the scholar community plays a crucial role, which must be accompanied by a robust policy within the school setting [50]. In this way, the school’s social environment is shown to be more determinant than other social variables such as SES and the family per se [21,51]. This is because children’s lower cognitive profile from a low SES seems to be affected by the lack of experiences (i.e., enriched environment) rather than permanent deficits in executive function development mechanisms [52]. Thereby, schools can apply diverse activities and experiences increasing physical fitness and cognitive skills, even simultaneously [53,54], which would reduce the detrimental impact of social inequalities and disadvantages on children and adolescents.

### 4.2. The Mediation Role of Fitness between SVI and Cognitive Performance

Our study contributed to three relevant findings related to the mediation role of fitness. The first, the global fitness score and all its components seemed to mediate the relationship between SVI and cognitive performance. Second, the three fitness components showed high interdependence; thereby, all of them and not only one contribute to the mediation role of the global fitness score. And third, the mediation role of fitness was significant only when high- and mid-SVI levels were compared to the low-SVI group but not between the highest two SVI levels (high vs. mid-SVI).

There are only a few studies in this research area, which makes possible comparisons difficult. In this sense, a structural equation model by Lemes et al. [45] involving this adolescent sample showed that SVI (same indicator used in the present study) was negatively and directly associated with the global cognitive performance and presented an indirect relationship with cognition mediated by the adolescent global fitness level [45]. Whereas the study by Andersen et al. [42], similar to our approach, states that physical fitness could mediate around 18% and 30% the association between SES (education or family income, respectively) and academic achievement in a Danish school cohort. 

We speculate that the mediation effect observed in our study (ranged 2.8% to 9.0%) was lower than that obtained by Andersen et al. [42] due to differences (a) at the social and economic level between countries (Denmark vs. Chile), (b) in predictors (education or family income vs school vulnerability), (c) on the outcome (academic achievements vs cognitive performance), and (d) on the mediator included (CRF vs a global fitness score). Overall, the study by Lemes et al. [45], Andersen et al., [42] and the present one highlight the crucial role of physical fitness in order to mitigate the influence of school vulnerability and social disadvantages over cognitive functioning and academic achievement, two crucial predictors of adulthood SES, mental health, and problem behaviour [5].

Regarding differences among SVI levels related to the mediation role of fitness, our findings align with the study by De Greeff et al. [41] in the academic field. This research showed a positive association between CRF with mathematics and spelling, moderated by children’s socioeconomic level. Similar findings were established by Pate et al. [20] in a large sample size of schoolchildren from the USA, showing that poverty moderates the association between CRF and academic achievement.

Supporting both findings, a study involving 1091 children and adolescents, which analysed brain cortical thickness through high-resolution magnetic resonance imaging technic, found that associations between cortical thickness and cognitive performance varied by SES for both language and executive function abilities [55]. Adding to this, it has been established that children and adolescents’ participation in sports and physical activity also depends on their SES [56]. However, it is also true that providing opportunities for exercising (improving the types and quality of resources) is an effective strategy to increase physical activity and reduce socioeconomic disparities [57]. 

Thus, early interventions reducing social gaps, especially in disadvantaged populations and involving schools could mitigate schoolchildren’s adverse cognitive performance [58,59]. Nowadays, implementing programs that promote physical fitness and cognitive development becomes even more relevant due to the potential impact of COVID-19 at social, economic, academic, and health levels [60,61,62], which could exacerbate the cognitive gap caused by social inequalities in children and adolescents.

### 4.3. Strengths and Limitations

This study contributes to the geographical gap in this research area, being to the best of our knowledge, the first in Latin America and worldwide evaluating the association combined and mediation role of physical fitness between a school vulnerability-related factor and cognitive performance in a large sample of adolescents. Thus, this study seems to be the first in showing a “narrowing” in the adolescent’s cognitive gap related to school vulnerability through a modifiable, low-cost, and practical factor, such as physical fitness. Moreover, our analyses include several covariates, but one of them, the BMIz, has been widely related to low cognitive and academic performance in children and adolescents [36,63]. In this way, this methodological approach adds more confidence to our findings, and it is crucial to mention because Chilean children and adolescents present one of the highest rates of overweight and obesity worldwide (54%) [64]. 

Another relevant point to highlight is that we have used two global scores related to fitness and cognition. This might be considered a strength due to the complexity of these measures that might be evaluated, ideally, considering the diversity of its components [52]. Hence, we conformed two robust and realistic measures because people generally have improved certain components over others (e.g., cardiorespiratory more than muscular strength, or working memory more than inhibitory control). Finally, the main limitation of the study was its cross-sectional approach, excluding causal inferences. Similarly, emotional variables such as motivation and self-esteem and the lack of a personal socioeconomic indicator must be considered to rule out residual confounding.

## 5. Conclusions

In conclusion, both SVI and physical fitness are powerful and decisive factors associated with adolescent cognitive performance. Additionally, the negative SVI relationship on cognitive performance seems to be ameliorated in adolescents with an adequate physical fitness level. This beneficial association is shown to be more evident in adolescents belonging to more unfavourable social backgrounds. In this sense, the present study shows that physical fitness and all its components (CRF, MF, and SAF) should be deemed as a protective social factor associated with narrowing the cognitive gap in adolescents linked to social vulnerability. This study calls public policymakers to encourage the government, school establishments, and parents to double efforts promoting physical activity, focusing on enhanced physical fitness as a primary outcome. This relatively low-cost and accessible measure could help in reducing social disadvantages and inequalities from the early stages; however, this assumption must be confirmed through future experimental studies.

## Figures and Tables

**Table 1:** Participants’ characteristics (mean ± SD) according to their school vulnerability level.

Participants’ Characteristics	All (n = 912)	Low-SVI (n = 246)	Mid-SVI (n = 252)	High-SVI (n = 414)	*p*-Value
Age (years)	11.7 ± 1.1	11.8 ± 1.0	11.5 ± 1.1	11.7 ± 1.1	<0.001 *
Sex (n = boys/n = girls)	460/452	110/136	126/126	224/190	0.151
Schools (n)	19 (912)	5 (246)	4 (252)	10 (414)	<0.001 *
Weight (kg)	50.0 ± 11.9	49.9 ± 11.2	47.9 ± 10.7	51.4 ± 12.7	0.006 *
Height (cm)	152.3 ± 9.4	154.1 ± 8.9	150.9 ± 8.8	152.1 ± 9.9	<0.001 *
BMI (z-score)	1.021 ± 1.1	0.826 ± 1.0	0.964 ± 1.0	1.172 ± 1.1	<0.001 *
PHV	−0.554 ± 1.2	0.273 ± 1.3	0.759 ± 1.2	0.596 ± 1.2	<0.001 *
Global fitness score	0.00 ± 3.1	1.27 ± 2.9	0.41 ± 2.8	0.49 ± 3.2	<0.001 *
CRF (z-score)	0.019 ± 1.0	0.281 ± 1.0	0.013 ± 0.9	0.202 ± 0.9	<0.001 *
MF (z-score)	0.039 ± 1.7	0.678 ± 1.6	0.043 ±1.6	0.291 ± 1.7	<0.001 *
SAF (z-score)	0.012 ± 1.0	0.311 ± 0.8	0.357 ± 1.0	0.00 ± 1.1	<0.001 *
Global cognitive performance	100.5 ± 8.8	104.0 ± 9.1	101.9 ± 8.0	97.6 ± 8.2	<0.001 *

SVI: School vulnerability Index; BMI: body mass index; PHV: Peak height velocity. CRF: cardiorespiratory fitness; MF: Muscular fitness; SAF: Speed/Agility fitness. Significant association: * *p* < 0.05.

**Table 2:** Zero-order correlations among principal study variables.

	School Vulnerability Levels	Global Cognitive Performance
Global fitness score	rho-0.206 *	r 0.208 *
CRF	rho-0.193 *	r 0.186 *
MF	rho-0.226 *	r 0.182 *
SAF	rho-0.065 *	r 0.151 *
Global cognitive performance	rho-0.321 *	--

Zero-order correlations (Pearson’s r or Spearman’s rho); CRF: cardiorespiratory fitness; MF: Muscular fitness; SAF: Speed/Agility fitness. Significant association: * *p* < 0.05.

## Data Availability

The data presented in this study are available on request from the corresponding author. The data are not publicly available due to ethical concerns.

## Supplementary Materials

The following are available online at https://www.mdpi.com/article/10.3390/ijerph181910073/s1. Table S1. Fitness mediation analyses on adolescents’ cognitive performance by SVI levels according the two models.

## References

[1] Cheng H., Furnham A. (2012). Childhood cognitive ability, education, and personality traits predict attainment in adult occupational prestige over 17 years. J. Vocat. Behav..

[2] Campbell F., Conti G., Heckman J.J., Moon S.H., Pinto R., Pungello E., Pan Y. (2014). Early childhood investments substantially boost adult health. Science.

[3] Caspi A., Houts R.M., Belsky D.W., Harrington H., Hogan S., Ramrakha S., Poulton R., Moffitt T.E. (2016). Childhood forecasting of a small segment of the population with large economic burden. Nat. Hum. Behav..

[4] Diamond A., Ling D.S. (2016). Conclusions about interventions, programs, and approaches for improving executive functions that appear justified and those that, despite much hype, do not. Dev. Cogn. Neurosci..

[5] Feinstein L., Bynner J. (2004). The importance of cognitive development in middle childhood for adulthood socioeconomic status, mental health, and problem behavior. Child Dev..

[6] Moffitt T.E., Arseneault L., Belsky D., Dickson N., Hancox R.J., Harrington H., Houts R., Poulton R., Roberts B.W., Ross S. (2011). A gradient of childhood self-control predicts health, wealth, and public safety. Proc. Natl. Acad. Sci. USA.

[7] Richmond-Rakerd L.S., Caspi A., Ambler A., d’Arbeloff T., de Bruine M., Elliott M., Harrington H., Hogan S., Houts R.M., Ireland D. (2021). Childhood self-control forecasts the pace of midlife aging and preparedness for old age. Proc. Natl. Acad. Sci. USA.

[8] Duncan G.J., Yeung W.J., Brooks-Gunn J., Smith J.R. (1998). How much does childhood poverty affect the life chances of children?. Am. Sociol. Rev..

[9] Rosen M.L., Hagen M.P., Lurie L.A., Miles Z.E., Sheridan M.A., Meltzoff A.N., McLaughlin K.A. (2020). Cognitive stimulation as a mechanism linking socioeconomic status with executive function: A longitudinal investigation. Child Dev..

[10] Singh A.S., Saliasi E., van den Berg V., Uijtdewilligen L., de Groot R.H.M., Jolles J., Andersen L.B., Bailey R., Chang Y.-K., Diamond A. (2019). Effects of physical activity interventions on cognitive and academic performance in children and adolescents: A novel combination of a systematic review and recommendations from an expert panel. Br. J. Sports Med..

[11] Schoentgen B., Gagliardi G., Défontaines B. (2020). Environmental and cognitive enrichment in childhood as protective factors in the adult and aging brain. Front. Psychol..

[12] Stillman C.M., Esteban-Cornejo I., Brown B., Bender C.M., Erickson K.I. (2020). Effects of exercise on brain and cognition across age groups and health states. Trends Neurosci..

[13] Chaddock L., Hillman C.H., Pontifex M.B., Johnson C.R., Raine L.B., Kramer A.F. (2012). Childhood aerobic fitness predicts cognitive performance one year later. J. Sports Sci..

[14] Van Waelvelde H., Vanden Wyngaert K., Mariën T., Baeyens D., Calders P. (2020). The relation between children’s aerobic fitness and executive functions: A systematic review. Infant Child Dev..

[15] Solis-Urra P., Sanchez-Martinez J., Olivares-Arancibia J., Castro Piñero J., Sadarangani K.P., Ferrari G., Rodríguez F., Gaya A., Fochesatto C.F., Cristi-Montero C. (2021). Physical fitness and its association with cognitive performance in Chilean schoolchildren: The Cogni-Action Project. Scand. J. Med. Sci. Sports.

[16] Åberg M.A., Pedersen N.L., Torén K., Svartengren M., Bäckstrand B., Johnsson T., Cooper-Kuhn C.M., Åberg N.D., Nilsson M., Kuhn H.G. (2009). Cardiovascular fitness is associated with cognition in young adulthood. Proc. Natl. Acad. Sci. USA.

[17] Fühner T., Kliegl R., Arntz F., Kriemler S., Granacher U. (2021). An Update on Secular Trends in Physical Fitness of Children and Adolescents from 1972 to 2015: A Systematic Review. Sports Med..

[18] Aran-Filippetti V., Richaud de Minzi M.C. (2012). A structural analysis of executive functions and socioeconomic status in school-age children: Cognitive factors as effect mediators. J. Genet. Psychol..

[19] González L., Cortés-Sancho R., Murcia M., Ballester F., Rebagliato M., Rodríguez-Bernal C.L. (2020). The role of parental social class, education and unemployment on child cognitive development. Gac. Sanit..

[20] Pate R.R., Clennin M., Shull E.R., Reed J.A., Dowda M. (2020). Poverty status moderates the relationship between cardiorespiratory fitness and academic achievement. J. Sch. Health.

[21] Flores-Mendoza C., Mansur-Alves M., Ardila R., Rosas R.D., Guerrero-Leiva M.K., Maqueo M.E.L.-G., Gallegos M., Colareta N.R., León A.B. (2015). Fluid intelligence and school performance and its relationship with social variables in Latin American samples. Intelligence.

[22] Solis-Urra P., Olivares-Arancibia J., Suarez-Cadenas E., Sanchez-Martinez J., Rodriguez-Rodriguez F., Ortega F.B., Esteban-Cornejo I., Cadenas-Sanchez C., Castro-Piñero J., Veloz A. (2019). Study protocol and rationale of the “Cogni-action project” a cross-sectional and randomized controlled trial about physical activity, brain health, cognition, and educational achievement in schoolchildren. BMC Pediatr..

[23] López V., Oyanedel J., Bilbao M., Torres J., Oyarzún D., Morales M., Ascorra P., Carrasco C. (2017). School achievement and performance in Chilean high schools: The mediating role of subjective wellbeing in school-related evaluations. Front. Psychol..

[24] Ruiz J.R., Castro-Pinero J., Espana-Romero V., Artero E.G., Ortega F.B., Cuenca M.M., Jimenez-Pavon D., Chillon P., Girela-Rejon M.J., Mora J. (2011). Field-based fitness assessment in young people: The ALPHA health-related fitness test battery for children and adolescents. Br. J. Sports Med..

[25] Tomkinson G.R., Lang J.J., Léger L.A., Olds T.S., Ortega F.B., Ruiz J.R., Tremblay M.S. (2019). Response to Criticisms of the 20 m Shuttle Run Test: Deflections, Distortions and Distractions. BJSM.

[26] Morrison G.E., Simone C.M., Ng N.F., Hardy J.L. (2015). Reliability and validity of the NeuroCognitive Performance Test, a web-based neuropsychological assessment. Front. Psychol..

[27] Moore S.A., McKay H.A., Macdonald H., Nettlefold L., Baxter-Jones A.D., Cameron N., Brasher P.M. (2015). Enhancing a somatic maturity prediction model. Med. Sci. Sports Exerc..

[28] Onis M.d., Onyango A.W., Borghi E., Siyam A., Nishida C., Siekmann J. (2007). Development of a WHO growth reference for school-aged children and adolescents. Bull. World Health Organ..

[29] Lumley T., Diehr P., Emerson S., Chen L. (2002). The importance of the normality assumption in large public health data sets. Annu. Rev. Public Health.

[30] Cohen J., Hillsdale N.J. (1988). The t Test for Means. Statistical Power Analysis for the Behavioural Sciences.

[31] Hayes A.F. (2017). Introduction to Mediation, Moderation, and Conditional Process Analysis: A Regression-Based Approach.

[32] Mora-Gonzalez J., Esteban-Cornejo I., Cadenas-Sanchez C., Migueles J.H., Rodriguez-Ayllon M., Molina-García P., Hillman C.H., Catena A., Pontifex M.B., Ortega F.B. (2019). Fitness, physical activity, working memory, and neuroelectric activity in children with overweight/obesity. Scand. J. Med. Sci. Sports.

[33] Loprinzi P.D., Kane C.J. (2015). Exercise and Cognitive Function: A Randomized Controlled Trial Examining Acute Exercise and Free-Living Physical Activity and Sedentary Effects. Mayo Clinic Proceedings.

[34] Alioto A., Conde K., Salazar-Villanea M., Moncada-Jimenez J., Cahn-Weiner D., Johnson D. (2019). C-20 Cardiorespiratory Fitness Predicts Processing Speed Performance in Urban Latin Americans. Arch. Clin. Neuropsychol..

[35] Mora-Gonzalez J., Esteban-Cornejo I., Solis-Urra P., Migueles J.H., Cadenas-Sanchez C., Molina-Garcia P., Rodriguez-Ayllon M., Hillman C.H., Catena A., Pontifex M.B. (2020). Fitness, physical activity, sedentary time, inhibitory control, and neuroelectric activity in children with overweight or obesity: The ActiveBrains project. Psychophysiology.

[36] Poh B.K., Lee S.T., Yeo G.S., Tang K.C., Afifah A.R.N., Hanisa A.S., Parikh P., Wong J.E., Ng A.L.O., Group S.S. (2019). Low socioeconomic status and severe obesity are linked to poor cognitive performance in Malaysian children. BMC Public Health.

[37] Mazzoni C.C., Stelzer F., Cervigni M.A., Martino P. (2014). Impacto de la pobreza en el desarrollo cognitivo: Un análisis teórico de dos factores mediadores. Liberabit.

[38] Laube C., van den Bos W., Fandakova Y. (2020). The relationship between pubertal hormones and brain plasticity: Implications for cognitive training in adolescence. Dev. Cogn. Neurosci..

[39] Herting M.M., Chu X. (2017). Exercise, cognition, and the adolescent brain. Birth Defects Res..

[40] Wheatley C., Wassenaar T., Salvan P., Beale N., Nichols T., Dawes H., Johansen-Berg H. (2020). Associations between fitness, physical activity and mental health in a community sample of young British adolescents: Baseline data from the Fit to Study trial. BMJ Open Sport Exerc. Med..

[41] de Greeff J.W., Hartman E., Mullender-Wijnsma M.J., Bosker R.J., Doolaard S., Visscher C. (2014). Physical fitness and academic performance in primary school children with and without a social disadvantage. Health Educ. Res..

[42] Andersen M.P., Valeri L., Starkopf L., Mortensen R.N., Sessa M., Kragholm K.H., Vardinghus-Nielsen H., Bøggild H., Lange T., Torp-Pedersen C. (2019). The mediating effect of pupils’ physical fitness on the relationship between family socioeconomic status and academic achievement in a danish school cohort. Sports Med..

[43] Ishihara T., Morita N., Nakajima T., Okita K., Yamatsu K., Sagawa M. (2018). Direct and indirect relationships of physical fitness, weight status, and learning duration to academic performance in Japanese schoolchildren. Eur. J. Sport Sci..

[44] Gil-Espinosa F.J., Chillón P., Fernández-García J.C., Cadenas-Sanchez C. (2020). Association of Physical Fitness with Intelligence and Academic Achievement in Adolescents. Int. J. Environ. Res. Public Health.

[45] Lemes V., Sadarangani K.P., Aguilar-Farias N., Rodríguez-Rodríguez F., Martins C.M.d.L., Felin Fochesatto C., Cristi-Montero C. (2021). Physical fitness plays a crucial mediator role in relationships among personal, social, and lifestyle factors with adolescents’ cognitive performance in a structural equation model. The Cogni-Action project. Front. Pediatr..

[46] Cadenas-Sanchez C., Migueles J.H., Esteban-Cornejo I., Mora-Gonzalez J., Henriksson P., Rodriguez-Ayllon M., Molina-García P., Löf M., Labayen I., Hillman C.H. (2020). Fitness, physical activity and academic achievement in overweight/obese children. J. Sports Sci..

[47] Ruotsalainen I., Gorbach T., Perkola J., Renvall V., Syväoja H.J., Tammelin T.H., Karvanen J., Parviainen T. (2020). Physical activity, aerobic fitness, and brain white matter: Their role for executive functions in adolescence. Dev. Cogn. Neurosci..

[48] Dutton E., van der Linden D., Lynn R. (2016). The negative Flynn Effect: A systematic literature review. Intelligence.

[49] Educación AdlCdl PISA 2018. Entrega de Resultados 2018. http://archivos.agenciaeducacion.cl/PISA_2018-Entrega_de_Resultados_Chile.pdf.

[50] Woods C.B., Volf K., Kelly L., Casey B., Gelius P., Messing S., Forberger S., Lakerveld J., Zukowska J., Bengoechea E.G. (2021). The evidence for the impact of policy on physical activity outcomes within the school setting: A systematic review. J. Sport Health Sci..

[51] OECD (2014). PISA 2012 Results: What Students Know and Can Do (Volume I, Revised edition, February 2014). https://www.oecd.org/pisa/keyfindings/pisa-2012-results-volume-I.pdf.

[52] Arán-Filippetti V. (2013). Structure and invariance of executive functioning tasks across socioeconomic status: Evidence from spanish-speaking children. Span. J. Psychol..

[53] Norris E., van Steen T., Direito A., Stamatakis E. (2020). Physically active lessons in schools and their impact on physical activity, educational, health and cognition outcomes: A systematic review and meta-analysis. Br. J. Sports Med..

[54] Doherty A., Forés Miravalles A. (2019). Physical Activity and Cognition: Inseparable in the Classroom. Front. Educ..

[55] Brito N., Piccolo L., Noble K. (2017). Associations between cortical thickness and neurocognitive skills during childhood vary by family socioeconomic factors. Brain Cogn..

[56] Bangsbo J., Krustrup P., Duda J., Hillman C., Andersen L.B., Weiss M., Williams C.A., Lintunen T., Green K., Hansen P.R. (2016). The Copenhagen Consensus Conference 2016: Children, youth, and physical activity in schools and during leisure time. Br. J. Sports Med..

[57] Moore L., Evenson K., McGinn A., Brines S. (2008). Availability of recreational resources in minority and low socioeconomic status areas (vol 34, pg 16, 2008). Am. J. Prev. Med..

[58] Christensen D.L., Schieve L.A., Devine O., Drews-Botsch C. (2014). Socioeconomic status, child enrichment factors, and cognitive performance among preschool-age children: Results from the Follow-Up of Growth and Development Experiences study. Res. Dev. Disabil..

[59] Fracchia C.S., Segretin M.S., Hermida M.J., Prats L.M., Lipina S.J. (2020). Mediating role of poverty in the association between environmental factors and cognitive performance in preschoolers. Rev. Argent. Cienc. Comport. (RACC).

[60] Douglas M., Katikireddi S.V., Taulbut M., McKee M., McCartney G. (2020). Mitigating the wider health effects of covid-19 pandemic response. BMJ.

[61] Nicola M., Alsafi Z., Sohrabi C., Kerwan A., Al-Jabir A., Iosifidis C., Agha R. (2020). The socio-economic implications of the coronavirus and COVID-19 pandemic: A review. Int. J. Surg..

[62] Kuhfeld M., Soland J., Tarasawa B., Johnson A., Ruzek E., Liu J. (2020). Projecting the potential impact of COVID-19 school closures on academic achievement. Educ. Res..

[63] Esteban-Cornejo I., Reilly J., Ortega F.B., Matusik P., Mazur A., Erhardt E., Forslund A., Vlachopapadopoulou E.A., Caroli M., Boyland E. (2020). Paediatric obesity and brain functioning: The role of physical activity—A novel and important expert opinion of the European Childhood Obesity Group. Pediatr. Obes..

[64] Junaeb (2021). Mapa Nutricional 2020.

