# Supplementary material for: Could Physical Fitness Be Considered as a Protective Social Factor Associated with Bridging the Cognitive Gap Related to School Vulnerability in Adolescents? The Cogni-Action Project
Source: Int J Environ Res Public Health. 2021 Sep 25;18(19):10073. doi: 10.3390/ijerph181910073 (PMC8507640; doi:10.3390/ijerph181910073)
Supplement: Supplementary file 1 [file ijerph-18-10073-s001.zip › ijerph-1377220-supplementary.pdf]

Supplementary Material

Table S1. Fitness mediation analyses on adolescents' cognitive performance by SVI levels according the two models.

**Global Fitness Score**		**Total effect (c)**	**Direct effect (c')**	**Indirect effect (a*b)**	**Mediation %**	**Mediation Type**
**SVI-GFS-COG**	Model 1	-3.12* (-3.79, -2.44)	-2.84* (-3.52, -2.16)	-0.27* (-0.44, -0.13)	9.0	Partial
Low/Mid-SVI	Model 1	-1.60 (-3.12, -0.08)	-0.86 (-2.39, 0.67)	-0.74* (-1.18, -0.38)	46.3	Full
Low/High-SVI	Model 1	-5.99* (-7.36, -4.61)	-5.32* (-6.70, -3.94)	-0.66* (-1.06, -0.34)	11.2	Partial
Mid/High-SVI	Model 1	-4.38* (-5.71, -3.05)	-4.46* (-5.77, -3.15)	0.07 (-0.12, 0.31)	-1.8	No mediation
**CRF**						
**SVI-CRF-COG**	Model 1	-3.23* (-3.89, -2.57)	-3.05* (-3.71, -2.38)	-0.18* (-0.32, -0.06)	5.6	Partial
	Model 2	-2.90* (-3.60, -2.23)	-2.85* (-3.54, -2.17)	-0.06 (-0.16, 0.00)	1.7	No mediation
Low/Mid-SVI	Model 1	-1.63* (-3.13, -0.13)	-1.38 (-2.87, 0.11)	-0.25* (-0.51, -0.06)	15.3	Full
	Model 2	-0.74 (-2.29, 0.81)	-0.78 (-2.33, 0.76)	0.04 (-0.05, 0.19)	-5.4	No mediation
Low/High-SVI	Model 1	-6.17* (-7.51, -4.83)	-5.79* (-7.14, -4.44)	-0.38* (-0.67, -0.14)	6.2	Partial
	Model 2	-5.47* (-6.85, -4.08)	-5.37* (-6.76, -3.99)	-0.09 (-0.27, 0.02)	1.8	No mediation
Mid/High-SVI	Model 1	-4.53* (-5.82, -3.25)	-4.41* (-5.68, -3.13)	-0.12 (-0.32, 0.02)	2.6	No mediation
	Model 2	-4.72* (-6.07, -3.38)	-4.59* (-5.94, -3.24)	-0.13 (-0.36, 0.02)	2.8	No mediation
**MF**						
**SVI-MF-COG**	Model 1	-3.25* (-3.91, -2.59)	-3.02* (-3.68, -2.35)	-0.23* (-0.40, -0.09)	7.1	Partial

	Model 2	-2.91* (-3.59, -2.23)	-2.85* (-3.54, -2.17)	-0.05 (-0.17, 0.03)	2.1	No mediation
Low/Mid-SVI	Model 1	-1.51* (-3.00, -0.02)	-1.11 (-2.60, 0.37)	-0.39* (-0.71, -0.14)	26.5	Full
	Model 2	-0.81 (-2.36, 0.74)	-0.78 (-2.33, 0.76)	-0.02 (-0.14, 0.04)	3.7	No mediation
Low/High-SVI	Model 1	-6.19* (-7.52, -4.86)	-5.67* (-7.03, -4.32)	-0.51* (-0.87, -0.22)	8.4	Partial
	Model 2	-5.47* (-6.85, -4.10)	-5.37* (-6.76, -3.99)	-0.10 (-0.31, 0.07)	1.8	No mediation
Mid/High-SVI	Model 1	-4.67* (-5.95, -3.40)	-4.56* (-5.83, -3.29)	-0.11 (-0.30, 0.03)	2.4	No mediation
	Model 2	-4.66* (-6.01, -3.32)	-4.59* (-5.94, -3.24)	-0.07 (-0.25, 0.05)	1.5	No mediation
**SAF**						
**SVI-SAF-COG**	Model 1	-3.27* (-3.92, -2.61)	-3.18* (-3.84, -2.53)	-0.08* (-0.17, -0.00)	2.8	Partial
	Model 2	-2.82* (-3.50, -2.13)	-2.85* (-3.54, -2.17)	0.03 (-0.00, 0.10)	-1.1	No mediation
Low/Mid-SVI	Model 1	-1.41 (-2.89, 0.06)	-0.66 (-2.16, 0.84)	-0.75* (-1.17, -0.38)	53.2	Full
	Model 2	-1.12 (-2.64, 0.40)	-0.78 (-2.33, 0.76)	-0.33* (-0.66, -0.05)	30.4	Full
Low/High-SVI	Model 1	-6.20* (-7.53, -4.86)	-5.88* (-7.20, -4.55)	-031* (-0.54, -0.13)	5.2	Partial
	Model 2	-5.34* (-6.73, -3.95)	-5.37* (-6.76, -3.99)	0.03 (-0.05, 0.15)	-0.6	Partial
Mid/High-SVI	Model 1	-4.78* (-6.05, -3.52)	-5.21* (-6.48, -3.95)	0.43* (0.18, 0.75)	-9.0	Partial
	Model 2	-4.22* (-5.53, -2.90)	-4.59* (-5.94, -3.24)	0.37* (0.07, 0.75)	-8.8	Partial

SVI: School Vulnerability Index, GFS: Global Fitness Score, COG: Cognitive performance, CRF: Cardiorespiratory fitness, MF: Muscular fitness, SAF: Speed/agility fitness. * indicates significative statistically. Model 1: adjusted for sex, PHV, BMIz, and schools. Model 2: Model 1 plus (other two fitness components).
